# Investigating Cooperative Behavior in Ecological Settings: An EEG Hyperscanning Study

**DOI:** 10.1371/journal.pone.0154236

**Published:** 2016-04-28

**Authors:** Jlenia Toppi, Gianluca Borghini, Manuela Petti, Eric J. He, Vittorio De Giusti, Bin He, Laura Astolfi, Fabio Babiloni

**Affiliations:** 1 Dept. of Computer, Control, and Management Engineering, "Sapienza" University of Rome, Via Ariosto 25, I – 00185, Rome, Italy; 2 IRCCS Fondazione Santa Lucia, Neuroelectrical Imaging and BCI Lab, Via Ardeatina 306, I– 00179, Rome, Italy; 3 Dept. of Molecular Medicine, "Sapienza" University of Rome, viale Regina Elena 291, Rome, Italy; 4 Dept. of Biomedical Engineering, University of Minnesota, 7–105 Hasselmo Hall 312 Church St. SE, Minneapolis, Minnesota, United States of America; 5 Carnegie Mellon University, Pittsburgh, PA 15213, Stati Uniti, United States of America; University of Electronic Science and Technology of China, CHINA

## Abstract

The coordinated interactions between individuals are fundamental for the success of the activities in some professional categories. We reported on brain-to-brain cooperative interactions between civil pilots during a simulated flight. We demonstrated for the first time how the combination of neuroelectrical hyperscanning and intersubject connectivity could provide indicators sensitive to the humans’ degree of synchronization under a highly demanding task performed in an ecological environment. Our results showed how intersubject connectivity was able to i) characterize the degree of cooperation between pilots in different phases of the flight, and ii) to highlight the role of specific brain macro areas in cooperative behavior. During the most cooperative flight phases pilots showed, in fact, dense patterns of interbrain connectivity, mainly linking frontal and parietal brain areas. On the contrary, the amount of interbrain connections went close to zero in the non-cooperative phase. The reliability of the interbrain connectivity patterns was verified by means of a baseline condition represented by formal couples, i.e. pilots paired offline for the connectivity analysis but not simultaneously recorded during the flight. Interbrain density was, in fact, significantly higher in real couples with respect to formal couples in the cooperative flight phases. All the achieved results demonstrated how the description of brain networks at the basis of cooperation could effectively benefit from a hyperscanning approach. Interbrain connectivity was, in fact, more informative in the investigation of cooperative behavior with respect to established EEG signal processing methodologies applied at a single subject level.

## Introduction

Studying the so-called “social brain” is one of the most emerging and challenging issues in neuroscience. Recent studies demonstrated how a deeper understanding of the neurophysiological basis of social behavior can be reached by analyzing two or more interacting people as a unique system [1–3]. The simultaneous recording of the cerebral activity from different subjects (“hyperscanning”) and the study of the synchronization between their brains were first proposed in fMRI as tools for the study of common vision [4] and social interaction [5,6] and then translated to neuroelectrical fields for the analysis of temporal motor synchronization [7–9], music production, [10–13], speech [14,15], shared attention [16] and decision making [17–20]. The introduction of the electroencephalographic (EEG) hyperscanning approach as well as the portable EEG recording system provided ecological experimental settings, whereby people are able to interact naturally [18,19,21,20]. This allowed the design of experiments in more realistic environments. The studies of EEG-based interbrain connectivity between subjects simultaneously engaged in a card game [19] or in tasks related to game theory [20,21] demonstrated the feasibility in predicting cooperative or defective behaviors before being overtly expressed.

The study of the neurophysiological bases of cooperative behavior is crucial for all of the applications in which the strict collaboration between two or more people is mandatory for the success of the task. This is the case, among many others, of commercial pilots, whose operational works require the exchange of information and the synchronization of their activities in order to successfully execute the flight mission. The safety of a flight, and thus of hundreds of people, is guaranteed by not only the individual pilot’s ability, but mainly by the cooperation of the crew as well. In fact, nowadays more than fifty percent of flight incidents and accidents are caused by human error. Since the 1979, *Crew Resource Management* (CRM) training has been widely used by many airline companies in order to teach pilots the right behavioral strategies to use during cooperative activities, and for the overall improvement of the safety of flights [22].

Despite the importance of crew cooperation, most of the literature of the last thirty years regarding aviation described cerebral and neuroelectrical aspects associated with the activities and abilities of single pilots. In particular, emphasis was focused on the use of neurophysiological signals for the assessment of mental workload, fatigue and drowsiness during flights [23–25], error management [26] and the prevention of accidents caused by pilots’ involuntary sleep [27,28]. It appears clear that the study of cerebral counterparts of efficient cooperation between pilots could open the way to a quantification of such “social interactions.” However, at the moment, this attempt has not been investigated in the literature due to the lack of technologies for recording cerebral activity in real operative environments, and neuroelectric indicators of synchronized or coordinated activities between team members.

In this paper, we proposed a combined approach of neuroelectrical hyperscanning and intersubject effective connectivity in order to: detect “cerebral signs of cooperation” between crews of professional pilots during a simulated flight in a full-motion simulator; provide neuroelectrical indicators capable of quantifying the degree of synchronization related to different levels of cooperation.

**Fig 1:**
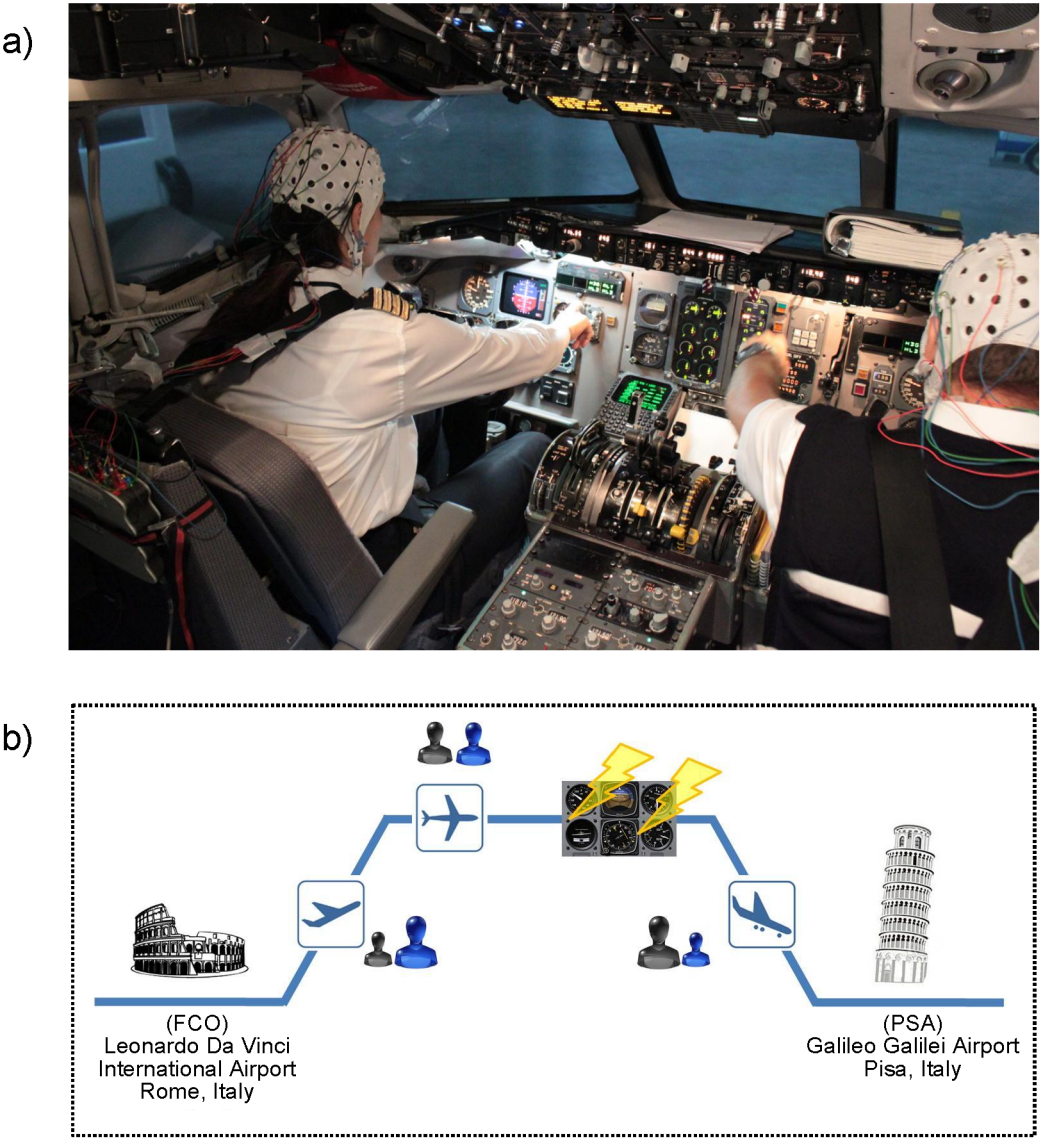
Experimental Design. a) Experimental setup in the simulator cockpit. b) Scheme of the flight performed by all crews. Pilots departed from Leonardo Da Vinci International Airport (FCO), Rome, Italy and the destination was the Galileo Galilei Airport (PSA), Pisa, Italy. During the cruise phase, an electrical failure was intentionally introduced to the Captain’s instrumentation in order to manipulate the regular execution of the flight. The level of interaction between the two crew members is represented by means of the icons located close to the flight phase symbol (blue → Captain; black → First Officer). The ratio between the size of the two icons describes the presence of an asymmetry in the interaction between pilots (takeoff: asymmetrical interaction between pilots unbalanced towards the CPT who has the control of the aircraft; cruise: symmetrical interaction between the two pilots; landing: asymmetrical interaction between pilots unbalanced towards the FO who took the control of the aircraft after the electrical failure to CPT’s instrumentation).

We collected behavioral, psychological, peripheral, and neurophysiological data simultaneously from pilot crews, *Captain* (CPT) and *First Officer* (FO), during a simulated flight (Fig 1a), in which workload and cooperation were modulated by inducing unexpected events during flight (Fig 1b). In particular, we simulated a specific electrical failure of the captain’s instrumentation in order to modify the *Pilot Flying* (PF) and *Pilot Not Flying* (PNF) roles of the crew within the flight. Such a role-exchanging contributed to create a non-standard situation requiring a higher degree of cooperation between the two pilots to complete the mission safely.

Behavioral and peripheral data were used to verify the capability of the ecological task in eliciting different levels of cooperation as desired during the experiment design.

To describe the neural basis of cognitive processes underlying different cooperative behaviors, we first characterized the different phases of the flight by means of spectral mapping, and an effective connectivity approach applied at a single subject level. Then, we computed a multi-subject analysis performed on pilots who were simultaneously recorded (real couples) and compared the results with those obtained by randomly mixing the pilots on the basis of the roles (formal couples).

The study of the pilots’ interbrain connectivity was performed on the different flight phases that required strong cooperation (such as takeoff or landing) as well as during those requiring a very low degree of cooperation (cruise). Such interbrain functional links returned information about the degree of synchronization between the brains across the different flight phases. A graph theory approach was then used to extract quantifiable indicators describing global and local properties of the cooperation networks.

Preliminary results about this study were reported in conference proceedings [29,30], where we provided, by means of a single couple, a proof of concept on the use of hyperscanning and interbrain connectivity in such ecological context. In the present manuscript, we enlarged the population included in the study, confirming what we achieved on the single couple. Moreover, we added a control condition, represented by couples of pilots randomly paired on the basis of the role (formal couples), for strengthening the results achieved for interbrain connectivity. A comparison between the interbrain patterns achieved in real and formal couples allowed to demonstrate the robustness of the approach to the detection of spurious links due to several factors and not directly related to the social aspects of the experiment. Additionally, we enriched the analysis through graph theory indices, providing biomarkers for social behavior.

## Methods

### Experimental Design

Six couples of civil pilots (age: 42.8±7.1, 11 males, 1 female) from the national Italian airline (Alitalia) participated in the study. In particular, the six CPTs had a total mean flight hours of 11000±2500, while the six FOs had a total mean flight hours of 5900±650 (mean±std). Written informed consent was obtained from the pilots after explanation of the study, which was approved by the local institutional ethics committee of Fondazione Santa Lucia Hospital. Each crewperson was asked to take part in a simulation test with a professional full-motion MD-80 simulator as a part of their continuous training in the airline company. Each crew was briefed by a qualified *Human Factor* (HF) Instructor from Alitalia about the aim of the mission and the technical details related to the experimental flight, including the execution of a *Brain-Computer-Interface* (BCI) task during the cruise phase. The test consisted of a commercial flight from the *Leonardo Da Vinci* International Airport (FCO) of Rome (Italy) to the *Galileo Galilei* Airport (PSA) of Pisa (Italy). The standard procedures of the MD-80 require that the CPT has to perform the takeoff (pilot flying–PF). During this phase, the CPT had to command the airplane (PF) and the FO had to check the cockpit instrumentation and reported all of the information (pilot not flying–PNF) requested by the CPT. When the airplane reached the cruise level (FL240), the pilots were asked to execute a BCI task. The BCI task consisted in moving upward or downward a cursor on a computer screen by modulating the sensorimotor EEG rhythm (μ-rhythm). The aim of controlling the cursor was to hit the targets placed on the top and on the bottom of the screen. In particular, the target on the top represented the “point”, and the target on the bottom represented the “line” of the International Morse code [31]. Successively, the BCI system encoded the targets into Morse symbols with the aim to generate the Morse message: “CQ SOS CU”. Such message was a 30 balanced symbols sequence, 15 points and 15 lines and it was based on a traditional radio communication code. The Pilots executed the BCI tasks in sequence, first the CPT and then the FO, in order to have always a Pilot with the control of the airplane during the cruise phase. In particular, while the CPT was engaged in the execution of the BCI task, the FO took the control of the aircraft, and when the CPT completed it, the pilots swapped their activities; the FO then did the BCI task while the CPT took control of the aircraft. After the cruise phase, a partial electrical failure was introduced to the CPT’s instrumentation in order to switch the roles PF/PNF of the pilots. As the CPT had no more instrumentation, the FO became PF and the CPT PNF. Pilots did not receive any information about possible failures or events that could occur during the flight. In fact, the partial electrical failure made the CPT unable to act properly as PF, which forced them to give control of the airplane to the FO. The electrical failure occurred at the same time from the beginning of the flight (after the cruise phase) for each of the crews. The flight duration was scheduled for fifty minutes, and the weather conditions were roughly cloudy with possible windshare (W/S). In particular, at the departure airport (FCO) the pilots received information in order to presage the possibility of W/S (WIND 180V190 15G25) with temperature and high spread but without W/S alert. At the arrival airport (PSA), the weather conditions were WIND 130 15G20, BRK4500 and visibility of 8 km.

Fig 1a showed a picture of the full-motion MD-80 flight simulator located at the Training Center in Fiumicino (Italy), where the experiments were performed. The examined flight phases were associated with a particular level and kind of interaction between the two pilots:

- *Takeoff phase*: the CPT had control of the airplane during takeoff and the FO had to report all of the information derived by checking the aircraft instrumentation;
- *Cruise phase*: the CPT performed a BCI task while the FO controlled the airplane. After the CPT finished the BCI task, the pilots switched roles and the FO performed the BCI task while the CPT controlled the airplane.
- *Landing phase*: due to a partial electrical failure at the CPT’s instrumentation, the pilots had to switch the PF/PNF roles and the FO had to take the control of the airplane until the landing was complete.

As the baseline for the analysis, the taxi phase was selected. During this phase, the airplane was moved through the taxiway toward the runway for the takeoff, and the pilots checked the airplane’s instrumentation and commands.

During the first and the last phases of the flight, the two pilots had to interact intensively in order to reach the common goal (a safe flight). This interaction happened in two different directions during takeoff and landing thanks to the introduction of the partial electrical failure, which allowed us to switch the role of the two pilots. The cruise phase was characterized, instead, by a lack of interaction between the crewmembers. A scheme of the flight is reported in Fig 1b. After the flight simulation, a debriefing was given to the crews. The NASA-TLX (Hart and Staveland, 1988) questionnaires, for the subjective evaluation of the perceived workload during the overall flight, and a difficulty perception Likert (Linkert, 1932) scale for each flight phase were then submitted to the crew.

### Simultaneous Multi-subject EEG Recordings

The neuroelectric hyperscanning recordings were performed with two 16-channel EEG acquisition systems (g.tec Medical Engineering GmbH, Austria—15 EEG + 1ECG channels, reference on both earlobes) placed behind the seats of the pilots inside the cockpit of the flight simulator. The physiological signals (EEG and ECG) were collected with a sampling frequency of 256 Hz. In order to delete the sources of variance between the two different EEG systems, the two amplifiers used for the hyperscanning recording were calibrated by means of two test signals, a sine wave and a squared wave, both of amplitude 100 μV and frequency 10 Hz. Differences in the amplitude of recorded test signals were deleted by adjusting the gains of the two amplifiers accordingly. Then, the amplifiers were synchronized through a syncing marker.

**Fig 2:**
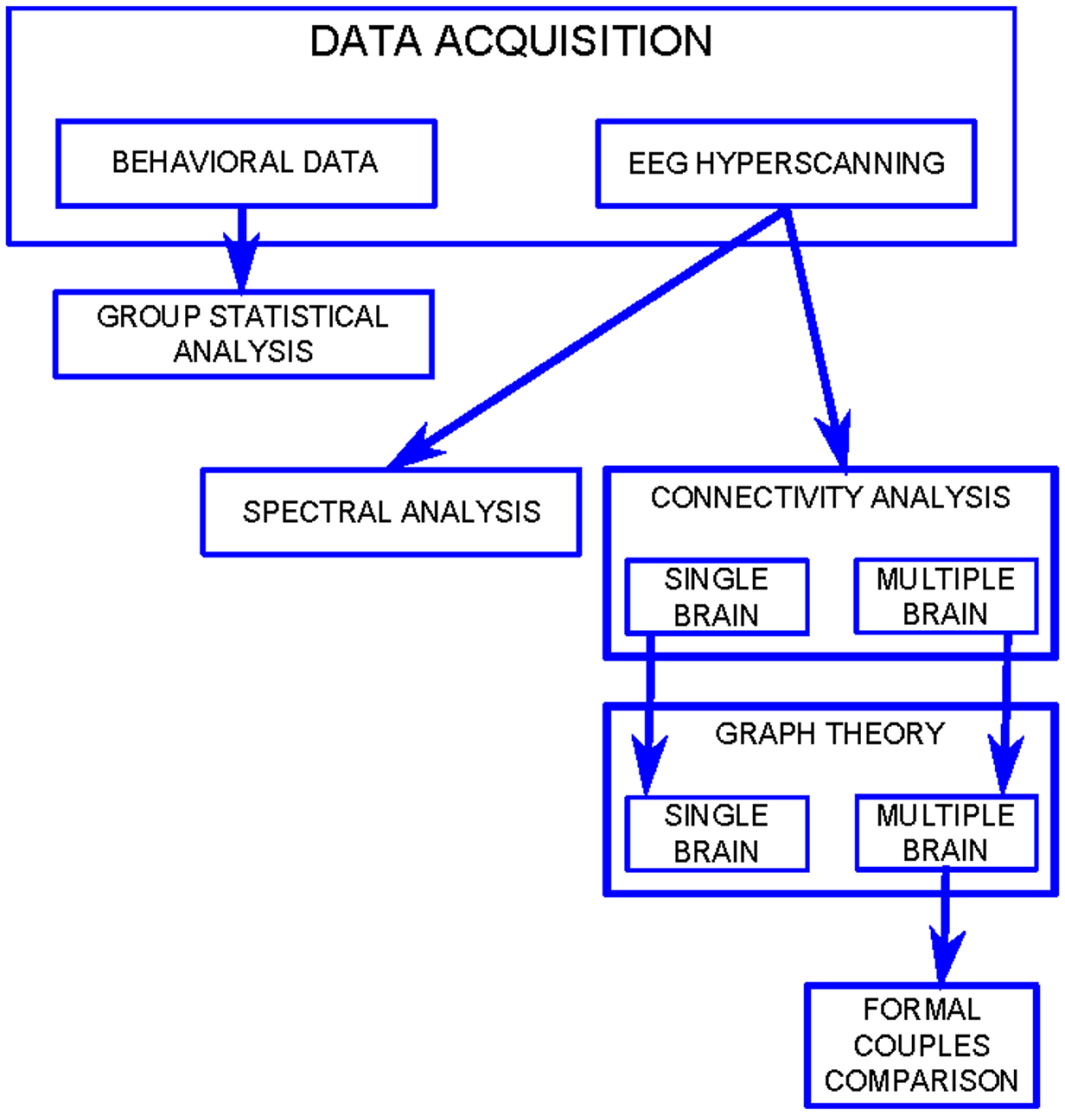
Flowchart. Flowchart summarizing data analysis process.

A flowchart of the data analysis is reported in Fig 2.

### Behavioral Data Analysis

At the end of the debriefing, the pilots were asked to fill out the NASA-TLX questionnaire for the subjective evaluation of the overall perceived workload and the Likert scale for the subjective evaluation of the perceived difficulty of the different flight phases. In particular, in the NASA-TLX, the pilots had to weigh (from 0 “very low” to 100 “very high”) and compare (which factor is more important than the others) six different workload factors (mental demand, physical demand, temporal demand, performance, effort, and frustration) in order to obtain a global score of the perceived workload. In the Likert scale, the pilots had to weigh (from 1 “very easy” to 5 “very difficult”) the different flight phases in order to have quantitative information of the perceived difficulty level for each of them. A statistical comparison (independent samples t-test, α = 0.05) between CPTs and FOs was computed for each score in order to detect differences between the two groups in the perception of workload and flight difficulty. The scores related to four flight phases (taxi, takeoff, cruise, and landing) were extracted for each pilot and subjected to a two-way repeated measure ANOVA considering the flight phases (FLIGHT-PHASE: taxi, takeoff, cruise, landing) as the within factor, and the two groups (ROLE: CPT, FO) as the between factor. Newman-Keuls’ post hoc tests were then applied to further investigate the significant factors.

### Autonomic Data Analysis

For the estimation of the Eye Blink Rate (EBR) and Heart Rate (HR) for each pilot and flight phase, the component of the eyes’ artifact (CEA), derived by the Independent Component Analysis (ICA) of the EEG signals, and the ECG signal were used. The CEA and the ECG signals were band-pass filtered (1–7 and 8–16 Hz, respectively) and then the eyes’ blink and R-peaks were found by applying a threshold criteria to the raw signals. The R-peaks were verified in terms of time-duration, amplitude and time-distance between consecutive R-peaks to rejects those due to artifacts. The EBR and HR were estimated by the following formula:

$$Rate=\left(\frac{fs}{ibi}\right)*60\;\left[\text{bpm}\right]$$ (1)

where *fs* is the sample frequency and the *ibi* (inter-beat-interval) is the distance between two consecutive R-peaks. As reported by Kaber et al. (2007), many researches have explored the use of HR for assessing operator workload in real time and to provide the basis for dynamic task allocation to automation or manual control [32–34]. These studies have revealed that an increased HR could be related to an increased mental workload. As the *Central Nervous System* (CNS) controls HR by varying the impulse traffic in the sympathetic and parasympathetic nerve fibers terminating in the sinoatrial node [35] an *HRindex* was defined as the linear combination of the HR and the arterial pressure (R-peak’s amplitude–HRamp). On the contrary, the EBR has been demonstrated to be inversely correlated with the increase of the mental workload [36].

The EBR, HR and HRamp values associated with the takeoff, cruise and landing phases were then normalized according to those achieved for the taxi phase (independent samples t-test, considering epochs as repetitions). The corresponding t-values were subjected to a two-way repeated measure ANOVA considering the flight phases (FLIGHT-PHASE: takeoff, cruise, landing) as the *within* factor, and the role (ROLE: CPT, FO) as the *between* factor. Newman-Keuls’ post hoc test was then applied to further investigate the significant factors.

### Neuroelectrical Data Analysis

EEG signals were band-pass filtered in the range 1–14 Hz in order to reduce the amount of muscular artifacts due to all the movements performed by the two pilots during the flight simulation. Then, *Independent Component Analysis* (ICA) was applied in order to remove eye-blinks artifacts [37] and the EEG traces were segmented according to the flight phases. In particular, we analyzed four segments of the mission: taxi, takeoff, cruise and landing. Then a semi-automatic procedure, based on threshold criterion (±80 μV), was applied in order to remove the residual artifacts. All the epochs in which at least one of the signals exceeded 80 μV in amplitude were excluded from the analysis. The epoch’s rejection rate was around fifty percent for each condition. Only the free-artifacts epochs in common for both pilots were considered in the further analyses (on average 50 epochs per condition). No statistical differences were found in the number of epochs between the different flight phases.

### Spectral Maps

Spectral activations at scalp level were reconstructed estimating the *Power Spectrum Density* (PSD) of the EEG signals recorded for each crew during all flight phases. PSD was averaged in two frequency bands: theta (3–7 Hz), alpha (8–13 Hz). In order to depict features common to each experimental group, we computed statistical spectral maps for each group (CPT/FO), for the three flight phases (takeoff, cruise and landing) and for each EEG frequency band. In particular, a paired t-test (considering N = 6 pilots as repetitions) with a significance level of 5% was computed on the PSD values achieved during the three flight phases and those extracted during the taxi condition. The test was conducted separately for the two experimental groups. False Discovery Rate (FDR) correction for multiple comparisons [38] was applied to statistical tests in order to reduce the occurrence of type I errors [39]. The group statistical spectral maps were obtained by representing on a 2D scalp model the significant corresponding t-values achieved in the statistical group analysis according to a predefined color scale. T-values under the significance threshold were mapped in grey.

### Single-subject Effective Connectivity

Effective connectivity was defined as the simplest cerebral circuit describing the causal relations experimentally observed between distinct signals recorded from different cerebral sites [40]. Among the different estimators defined in the context of effective connectivity, we selected those based on the concept of *Granger Causality* [41]. In fact, such an estimator unlike the *Structural Equation Modeling* [42] and *Dynamic Causal Model* [43], does not require any a priori knowledge about the connectivity structure and can thus be used when no specification about the connectivity linkages is available (exploratory tools) [44]. *Partial Directed Coherence* (PDC) is a GC based spectral estimator providing the directed influences between any given pair of signals in a multivariate data set [45,46]. Several studies have demonstrated the higher efficiency of approaches based on the use of *multivariate autoregressive* (MVAR) models built on original time-series, being the bivariate approach affected by a high number of false positives due to the impossibility of the method in discarding a common effect on a couple of signals of a third one acquired simultaneously. The PDC is also of particular interest because it can distinguish between direct and indirect connectivity flows in the estimated connectivity pattern better than the other multivariate GC approaches, the Directed Transfer Function [47] and its direct modified version [48].

The estimation of cerebral networks underlying the cognitive processes elicited by piloting an aircraft was performed for each pilot and each flight phase by computing PDC on EEG signals recorded at the scalp level during the flight simulation. The estimated patterns were statistically validated (significance level of 5% adjusted by means of FDR for multiple comparisons) against the null case by means of asymptotic statistic procedure [49,50] in order to discard all of the spurious connections due to random oscillations of EEG signals. Successively, such estimated patterns were averaged in two bands of interest: theta, on average 3–7 Hz and alpha, on average 8–13 Hz.

To describe and quantify the connectivity patterns estimated at single subject level and to summarize the network properties at a group level, we considered indices derived from graph theory [51]. The statistical approach described here allowed us to extract adjacency matrices for the computation of graph indices, avoiding the alterations of the real topological properties of the networks that may arise applying empirical approaches as demonstrated previously [52].

The adjacency matrix is built by comparing, for each couple of nodes and for each frequency sample, the achieved connectivity matrix with the corresponding statistical threshold computed by means of Asymptotic Statistic assessment method. In particular:

$$G_{ij}=\{\begin{array}{c} \;1\;\to \;\;A_{ij}\geq \;\tau_{ij}\\ \;0\;\to \;\;A_{ij}<\;\tau_{ij} \end{array}$$ (2)

where *G*_*ij*_ and *A*_*ij*_ represent the entry *(i*,*j)* of the adjacency matrix *G* and the connectivity matrix *A*, respectively, and *τ*_*ij*_ is the corresponding statistical threshold. The higher reliability of the statistical approach for extracting the adjacency matrix has been demonstrated [52] where a detailed comparison with the empirical methods is provided.

Among the several indices available nowadays, we selected three indices characterizing the global structure of the network (density, global efficiency, and local efficiency) and an index (anterior degree) characterizing the involvement of frontal regions of the scalp, which were demonstrated to be highly involved in tasks such as those analyzed in the present paper.

*Density* is the more general property of the network. It is defined as the number of significant connections divided by the total number of possible connections [51]:

$$den=\frac{L}{N\left(N-1\right)}$$ (3)

where *L* is the number of significant connections returned by the statistical assessment and *N* is the number of electrodes.

*Global Efficiency* is defined as the average of the inverse of the *geodesic length* and represents the efficiency of the communication between all the nodes in the network [53]:

$$E_{g}=\frac{1}{N\left(N-1\right)}\sum_{i\neq j}\frac{1}{d_{ij}}$$ (4)

where *d*_*ij*_ is the geodesic distance between nodes *i* and *j*.

*Local Efficiency* is defined as the average of the global efficiencies computed on each sub-graph belonging to the network and represents the efficiency of communication between all nodes around node *i* in the network [53]:

$$E_{l}=\frac{1}{N}\sum_{i=1}^{N}E_{g}\left(S_{i}\right)$$ (5)

where *N* represents the number of nodes in the graph and *S*_*i*_ the sub-graph achieved by deleting the *i*^th^ row and the *i*^th^ column from the original graph.

*Anterior Degree* is a measure of the involvement of the frontal areas of the scalp and is defined as the average of the frontal electrodes degree. The degree of a node is the number of links connected directly to it. In particular, it can be defined as:

degAnt=∑i=1NantwiNant (6)

$$w_{f}=\sum_{j\in N,j\neq f}G_{fj}+\sum_{i\in N,i\neq f}G_{if}$$ (7)

where *N*_*ant*_ represents the number of anterior electrodes (Fpz, AF3, AF4, F3, Fz, F4).

All of the graph indices were separately subjected to a two-way repeated measures ANOVA considering the flight phases (FLIGHT-PHASE: takeoff, cruise, landing) as the *within* factor, and the role (ROLE: CPT, FO) as the *between* factor. Newman-Keuls’ post hoc test was then applied to further investigate the significant factors.

### Intersubject Effective Connectivity

The estimation of the intersubject connectivity networks has been computed by following all of the steps described for the single subject approach. The only difference consisted in the construction of the MVAR model as base of the PDC estimation. The intersubject connectivity was performed, in fact, by generating a unique MVAR model based on the EEG data from two pilots belonging to the same crew. However, due to the fact that the intrinsic characteristics of EEG signals could show a high variability from subject to subject, a normalization procedure was applied. Data coming from each pilot in the couple were normalized according to the corresponding taxi condition. Such normalization allowed for one side to avoid spurious results due to differences in the amplitude of the two pilots in a couple and from the other side to discard all the intersubject links due to the exposure in the same experimental environment. The baseline corresponded, in fact, to a period in which the two pilots were exposed to the same external stimuli (monitors, instruments, etc.), without any interaction between them.

A sub-set of channels (F3, Fz, F4, C3, C4, P3, Pz, P4) among the sixteen recorded was selected for each pilot. In fact, the reduction of the dimension of the MVAR model used in the estimate led to an increase of the accuracy in the estimation process. After the construction of a unique MVAR model, including the simultaneous data of both pilots, effective connectivity was estimated by means of PDC and then validated through asymptotic statistics, imposing a significance level of 5% FDR corrected for multiple comparisons. Finally, the validated PDC values were averaged in two bands of interest: theta, on average 3–7 Hz, and alpha, on average 8–13 Hz, and then mapped on a scalp model.

As executed for single subject connectivity, some graph indices were extracted in order to statistically quantify the main properties of the intersubject networks. In particular, we considered indices describing the entity and the direction of the interaction between crew members in the different flight phases and also an index highlighting which cerebral areas of the two pilots operating in couples were highly interconnected during all of the cooperation phases. Such indices were computed for each couple, each flight phase, and for each frequency band.

The extension of the traditional methods for connectivity estimation to the multi-subject case led to the necessity of adapting the current graph indices definition to the description of the intersubject networks. The adjacency matrix achieved after the estimation and validation processes can be considered as composed by four different blocks. The block composed by the first *N* columns and the first *N* rows describes the connections within the first subject. The block composed by the last *N* columns and the last *N* rows describes the connections within the second subject. The block composed by the first *N* rows and the last *N* column describes the connection directed from the first to the second subject. The block composed by the last *N* rows and the first *N* columns includes the connection directed from the second to the first subject.

*Inter-connections Density* describes the entity of the interaction between the subjects. In particular, it is possible to define the interconnections density (D_IC_) as follows:

$$D_{IC}=\frac{\sum_{i=1}^{N}\sum_{j=N+1}^{2N}A_{ij}+\sum_{i=N+1}^{2N}\sum_{j=1}^{N}A_{ij}}{2N}$$ (8)

where *N* represents the number of signals included in the MVAR for each subject and *A*_*ij*_ the global adjacency matrix.

Such an index was subjected to a one-way repeated measures ANOVA considering the flight phases (FLIGHT-PHASE: takeoff, cruise, landing) as the within factor. Newman-Keuls’ post hoc test was then applied to further investigate the significant factors.

*Directed Density* allows us to distinguish the direction of the interaction. In particular:

$$D_{1\to 2}=\frac{\sum_{i=1}^{N}\sum_{j=N+1}^{2N}A_{ij}}{N}$$ (9)

$$D_{2\to 1}=\frac{\sum_{i=N+1}^{2N}\sum_{j=1}^{N}A_{ij}}{N}$$ (10)

where *D*_1→2_ describes the density of connections directed from the first to the second subject and *D*_2→1_ describes the density of connections directed from the second to the first subject.

A paired t-test (significance level of 5%) was applied to each of these two indices in order to compare the entity and the direction of interaction between the pilots during the two cooperative takeoff and landing phases. The work hypothesis is to investigate if the inversion of the role between CPT and FO due to the electrical failure could have effect on the intersubject interaction detected at neuroelectrical level.

*Inter-areas Links Density* is defined as the number of existing links connecting two cerebral areas over the number of possible links connecting them, and allows us to highlight which cerebral areas of the two pilots operating in couples were highly interconnected during all of the cooperation phases. In particular, the density of links connecting area *A*_*1*_ in the first pilot with area *A*_*2*_ in the second pilots can be defined as follows:

$$LD_{A_{1}A_{2}}=\frac{L_{A_{1}A_{2}}}{Ltot_{A_{1}A_{2}}}$$ (11)

where $L_{A_{1}A_{2}}$ is the number of links connecting the area *A*_*1*_ in the first pilot with the area *A*_*2*_ in the second pilots and $Ltot_{A_{1}A_{2}}$ represents the number of all possible links connecting the two areas. In the present paper, such index was computed between the frontal (F), central (C) and parietal (P) areas. Such index was subjected to a paired t-test (significance level of 5%) in order to evaluate differences in the CPT-FO interconnected cerebral areas between takeoff and landing.

In order to distinguish the real cooperative behavior from social interactions solely due to the role established for the two pilots within a couple, we repeated the same analysis on newly constructed couples in which pilots were paired only on the basis of their role (formal couples). In particular, each CPT was paired with all of the other FOs not directly involved with them during the experiment (15 possible couples). For each of the fifteen formal couples, intersubject connectivity networks were first estimated by means of PDC and then statistically validated against the null case. The achieved adjacency matrices were then used for extracting the interconnections density, demonstrated to be a measure of the level of cooperation between two interacting pilots. Formal couples represented a control condition for the PDC estimator since they allowed to reconstruct the intersubject connectivity pattern due not to a real social interaction but to the environment, to the role and to the exposition to common stimuli for the two pilots. All the differences, in terms of both patterns and indices, between real and formal couples were supposed to be strictly related to the social components of the task.

Interconnections Density was then subjected to a two-way repeated measures ANOVA, computed considering the flight phases (FLIGHT-PHASE: takeoff, cruise, landing) as the within factor and the couple type (COUPLE: real, formal) as the between factor. Newman-Keuls’ post hoc test was then applied to further investigate the significant factors.

## Results

### Psychological Tests

**Fig 3:**
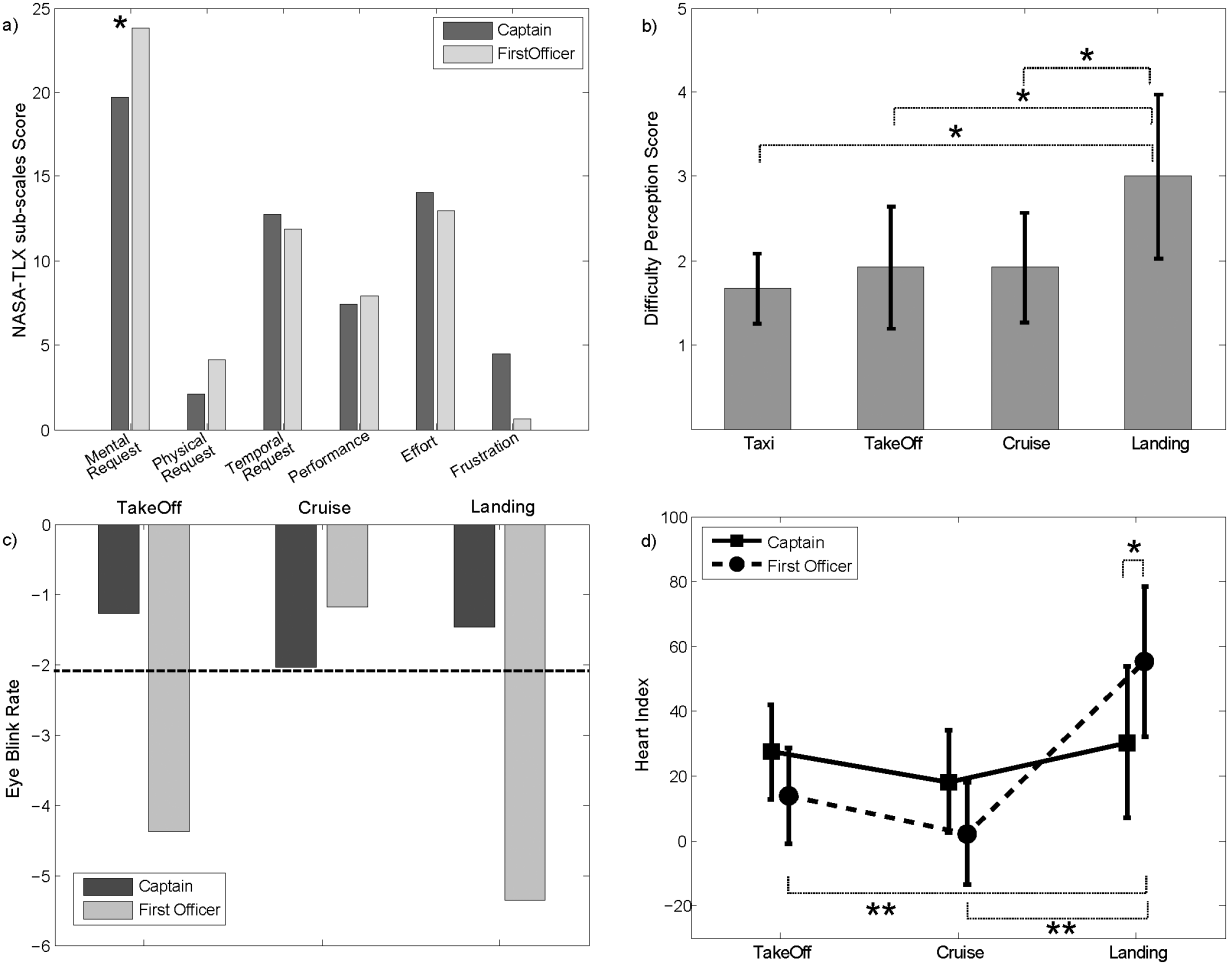
Behavioral Data Results. a) Bar diagram reporting the NASA-TLX sub-scale scores separately for Captains (dark grey) and First Officers (light grey). b) Results of the ANOVA were computed considering as a dependent variable the difficulty perception score and as *within* factor the FLIGHT PHASES (taxi, takeoff, cruise and landing). The bar diagram shows the mean value for the score assigned to each flight phase by all of the pilots (irrespective of the role). The bars represent their relative 95% confidence intervals. The symbol (*) indicates a statistical difference (Newman-Keuls’ pairwise comparisons) between the flight phases (p<0.05). c) Bar diagram reporting the t-values computed, separately for Captains (dark grey) and First Officers (light grey), on EBR for each flight phase (takeoff, cruise, landing), considering the taxi phase as a reference. The dotted black line represents the statistical threshold for the t-values. d) Diagram reporting the t-values computed, separately for Captains (solid line) and First Officers (dotted line), on the HR-Index for each flight phase (takeoff, cruise and landing) considering the taxi phase as a reference. The symbols (*) and (**) indicate a statistical difference (Newman-Keuls’ post hoc test) between the flight phases and the two pilots, respectively.

Fig 3a showed the mean values of the six sub-scales of the NASA-TLX, separately for the captains (CPTs–dark gray) and for the first officers (FOs–light gray). While the differences between CPTs and FOs in terms of global workload were not significant, it is possible to note how the FOs perceived higher mental demand than the CPTs (p = 0.038). The scores related to the four flight phases (taxi, takeoff, cruise, landing) were subjected to a two-way repeated measures ANOVA (within factor: flight phase; between factor: role). The results of the ANOVA revealed a significant influence of the FLIGHT-PHASE factor (F = 4.35, p = 0.0117) on the Difficulty Perception Score. No effects resulted, instead, for the ROLE factor and its interaction ROLE x FLIGHT-PHASE. Fig 3b showed the trend of Difficulty Perception Score, returned by the Likert scale, along the different flight phases. Before the electrical failure, the difficulty perceived for the different flight phases did not change among the different phases; whilst in the landing, the perception of the difficulty increased significantly, as confirmed by Newman-Keuls’ post hoc test.

### Peripheral Data

By the analysis of the ECG signal and the eye-blink component, the autonomic parameters of the Heart Rate Index (linear combination of the HR and the arterial pressure–HRindex) and Eye Blink Rate (EBR) were estimated for each flight phase and pilot. Results of the ANOVA on the EBR for the CPTs and for the FOs did not return any significant effect of the factors FLIGHT-PHASE and ROLE. However, a t-test between the EBR during each flight phase and the taxi returned significant differences for takeoff and landing (Fig 3c). CPTs kept the EBR almost constant across the considered flight phases, while the CPTs showed a significant reduction of the EBR during the takeoff and landing phase. In fact, during such flight phases, the FOs had to check all the information coming from the cockpit instruments, especially during the Landing. Due to the electrical failure, only the FO side was functioning, thus they had to pay lot of attention to the aircraft parameters provided by the instruments and, as consequence, the FOs showed a lower EBR than the CPTs.

The HR rate activity during different phases of the simulated flight revealed a significant variation of the HRindex according to the within factor FLIGHT-PHASE (F = 8.77, p = 0.00184) and to the interaction factor FLIGHT-PHASE x ROLE (F = 4.17, p = 0.031). The results for the interaction factor are reported in Fig 3d. As for the FOs, the HRindex was significantly higher during the landing with respect to the other phases. Moreover, the HRindex of the CPT and of the FO differed significantly only in the landing phase.

### Spectral Analysis

**Fig 4:**
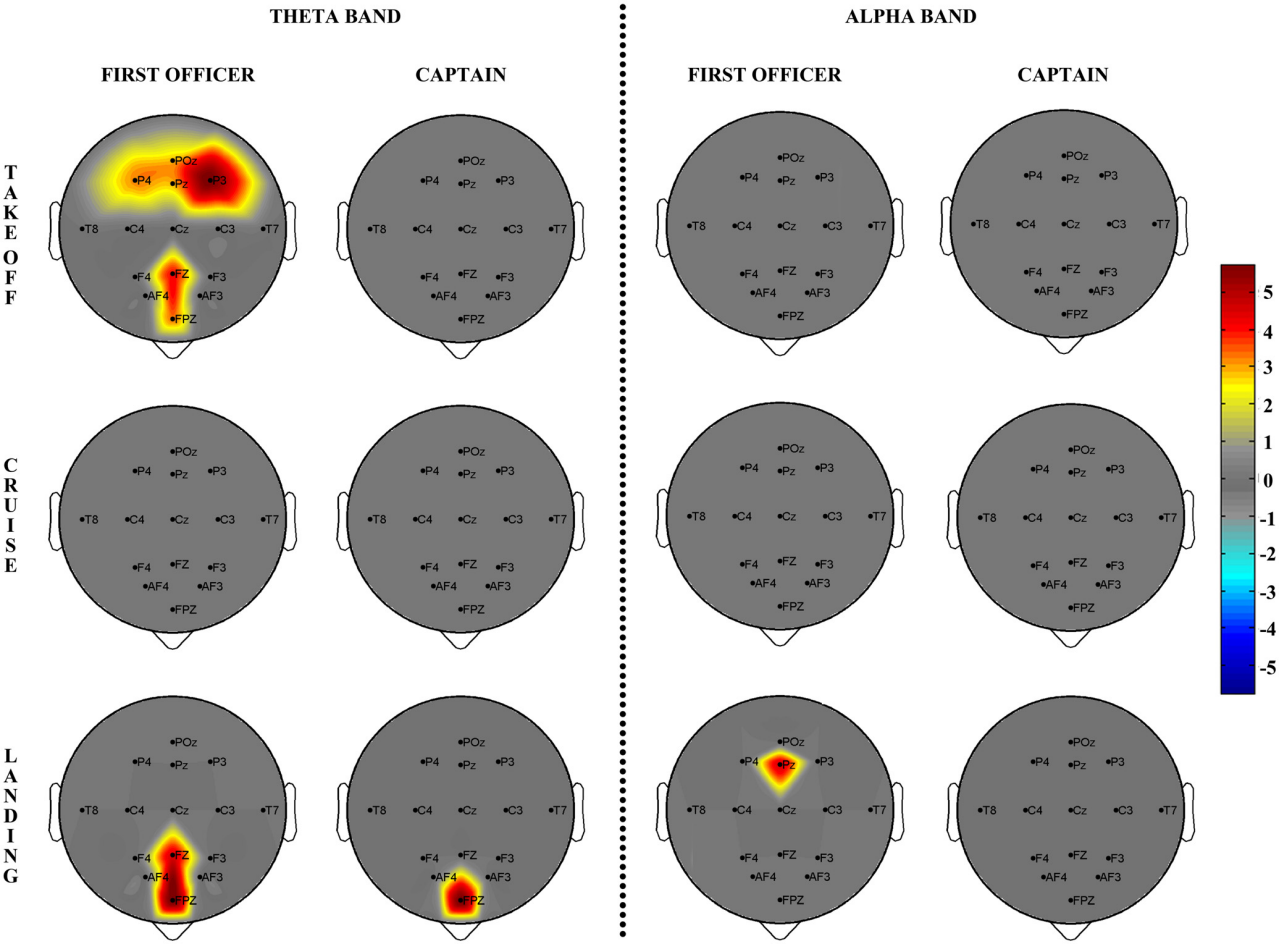
Spectral Maps. a) Group statistical spectral scalp maps (N = 6) computed, separately for Captains (second and fourth columns) and First Officers (first and third columns), for each of the three flight phases (takeoff, cruise and landing) in the theta and alpha EEG bands. Rendering of the scalp model is viewed from the above (nose pointing downward). The color of each pixel codes for the t-value achieved, for the related electrode, by comparing the spectral activity elicited during each flight phase with respect to the taxi phase (color scale on the right). All of the t-values below the statistical threshold are reported in grey.

Spectral activations at scalp level were reconstructed estimating *Power Spectrum Density* (PSD) of the EEG signals (16 channels) recorded during the three flight phases (takeoff, cruise, landing). The taxi phase was also recorded as the baseline. The group statistical spectral maps, for the theta 3–7 Hz and the alpha 8–13 Hz frequency bands, were obtained by representing on a 2D scalp model the significant t-values achieved for each of the electrodes by the statistical group analysis. Beta and gamma bands were excluded due to the high number of artifacts. T-values under the significance threshold (p<0.05) were mapped in grey. The results of the statistical spectral mapping performed separately for the two groups (CPT and FO) are reported in Fig 4. As for the CPT group, the only significant differences with respect to the taxi phase were found for frontal areas in the theta band during the landing phase. The FO group showed instead a significant spectral activation of frontal and parietal areas during the takeoff phase in the theta band. During the landing phase, FOs showed frontal brain activations in the theta band (similar to what was found in the CPT group) and parietal brain activations in the alpha band.

### Single-subject Effective Connectivity

Effective connectivity networks were estimated at a single subject level for each flight phase and frequency band and then characterized by means of graph indices. A repeated measures ANOVA was conducted for investigating the effect of both the FLIGHT-PHASE and ROLE factors on the properties of the networks elicited during the flight. In the theta band, we found a significant influence of the FLIGHT-PHASE factor on Density, Anterior Degree, Global Efficiency and Local Efficiency indices (Table 1). No significant results were found for the alpha band, even if a trend similar to that of the theta band was found. All four of the considered indices (density, anterior degree, local and global efficiency) showed a similar trend according to the three flight phases. Newman-Keuls’ pairwise comparisons revealed a significantly lower value of such indices during the cruise phase with respect to takeoff and landing, which did not show any statistical differences to each other. The networks elicited by the two pilots were thus more efficient and were characterized by a higher involvement of the frontal brain regions during takeoff and landing.

**Table 1:** Single-subject Connectivity Results.

	FLIGHT-PHASE		ROLE		FLIGHT-PHASE x ROLE	
Indices	F(2,20)	p	F(1,10)	p	F(2,20)	p
Density	**8.44**	**0.0022**	1.80	0.209	1.55	0.236
Anterior Degree	**6.71**	**0.0059**	2.48	0.146	1.26	0.305
Global Efficiency	**5.41**	**0.013**	1.50	0.248	1.38	0.275
Local Efficiency	**4.08**	**0.033**	1.91	0.197	1.93	0.171

Results of the ANOVA computed considering as main *within* factors FLIGHT-PHASE and ROLE and as dependent variables the four graph indices (Density, Anterior Degree, Global Efficiency, Local Efficiency) extracted for each single subject in theta band.

### Intersubject Effective Connectivity

**Fig 5:**
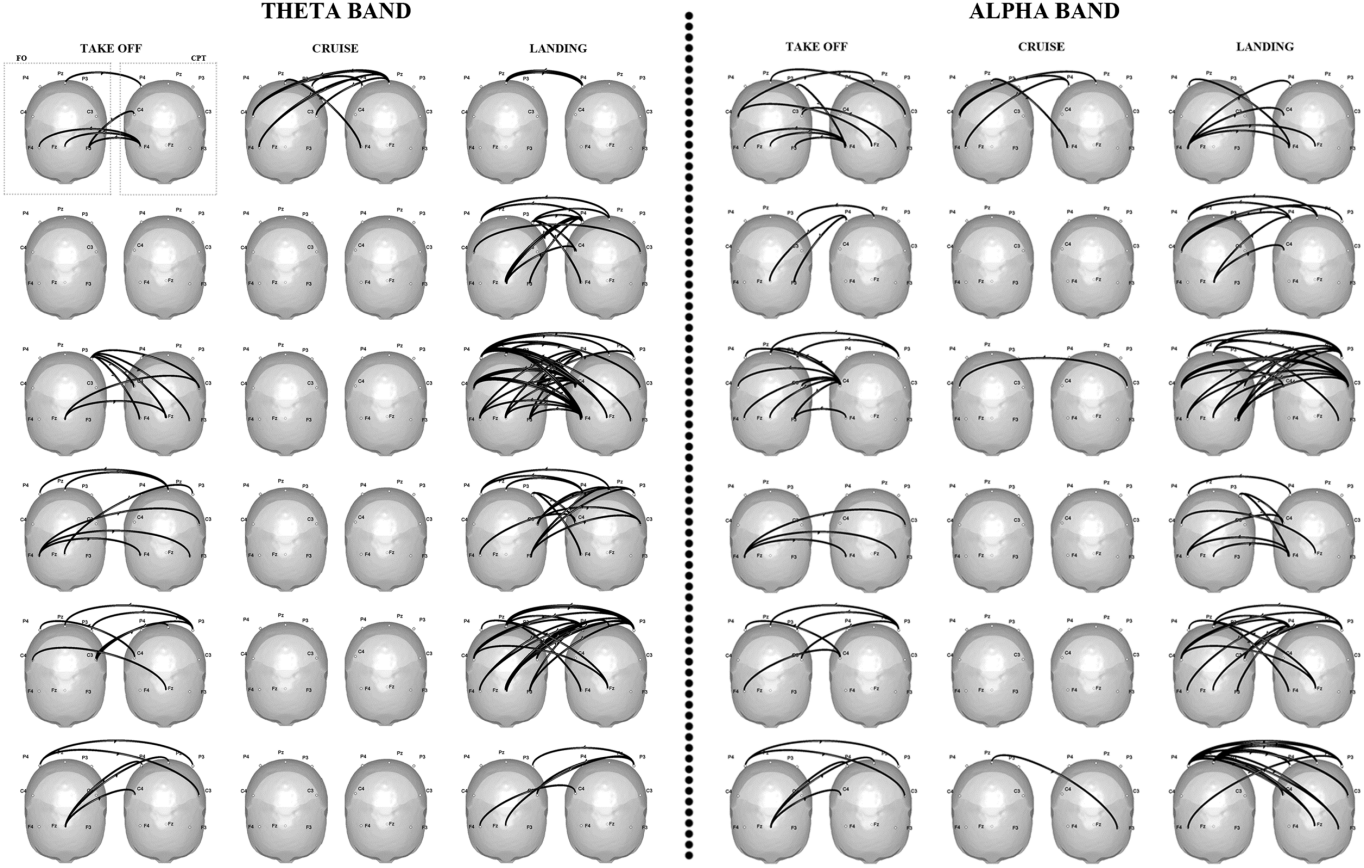
Inter-brain connectivity patterns. Statistically significant connectivity patterns elicited in the theta (3–7 Hz; panel a) and alpha bands (8–13 Hz; panel b) during the takeoff, cruise and landing phases by each pilot couple involved in the study (one couple for each row). The 3D scalp is seen from the above with the nose pointing toward the bottom of the page. Statistical connections between the two pilots’ brains activities are represented by means of black arrows. In the representation of each couple, the first and the second head correspond to the First Officer (FO) and to the Captain (CPT) respectively.

The results of the interbrain connectivity analysis were reported in Fig 5 for each of the six couples of pilots involved in the experiment. The connectivity patterns were achieved on the z-score (Zhang et al., 1999) values computed for each phase with respect to the taxi condition. All of the couples except one showed a denser pattern of interconnections linking the two brains’ activities during the two cooperative flight phases, with respect to the cruise phase (no cooperative phase). Such results were confirmed in both the theta and alpha bands.

To statistically confirm such density modulation according to the degree of cooperation between the two pilots, we defined an index (Inter-connections Density) quantifying the number of links exchanged between the two crew members. Such index was then subjected to ANOVA to evaluate its ability in discriminating the different levels of cooperation during the flight. The results of the ANOVA showed a significant effect of the main FLIGHT-PHASE within factor in both the theta (F = 4.57, p = 0.039) and alpha (F = 12.186, p = 0.00208) bands. Newman-Keuls’ post hoc analysis revealed a significantly higher number of inter-connections in the theta band during landing with respect to the other two flight phases. In the alpha band, the lowest number of interconnections resulted during the cruise phase, confirming what is qualitatively shown by Fig 5. Takeoff and landing were characterized by a higher number of interconnections with respect to the cruise phase, with a prevalence of connections in the landing.

**Fig 6:**
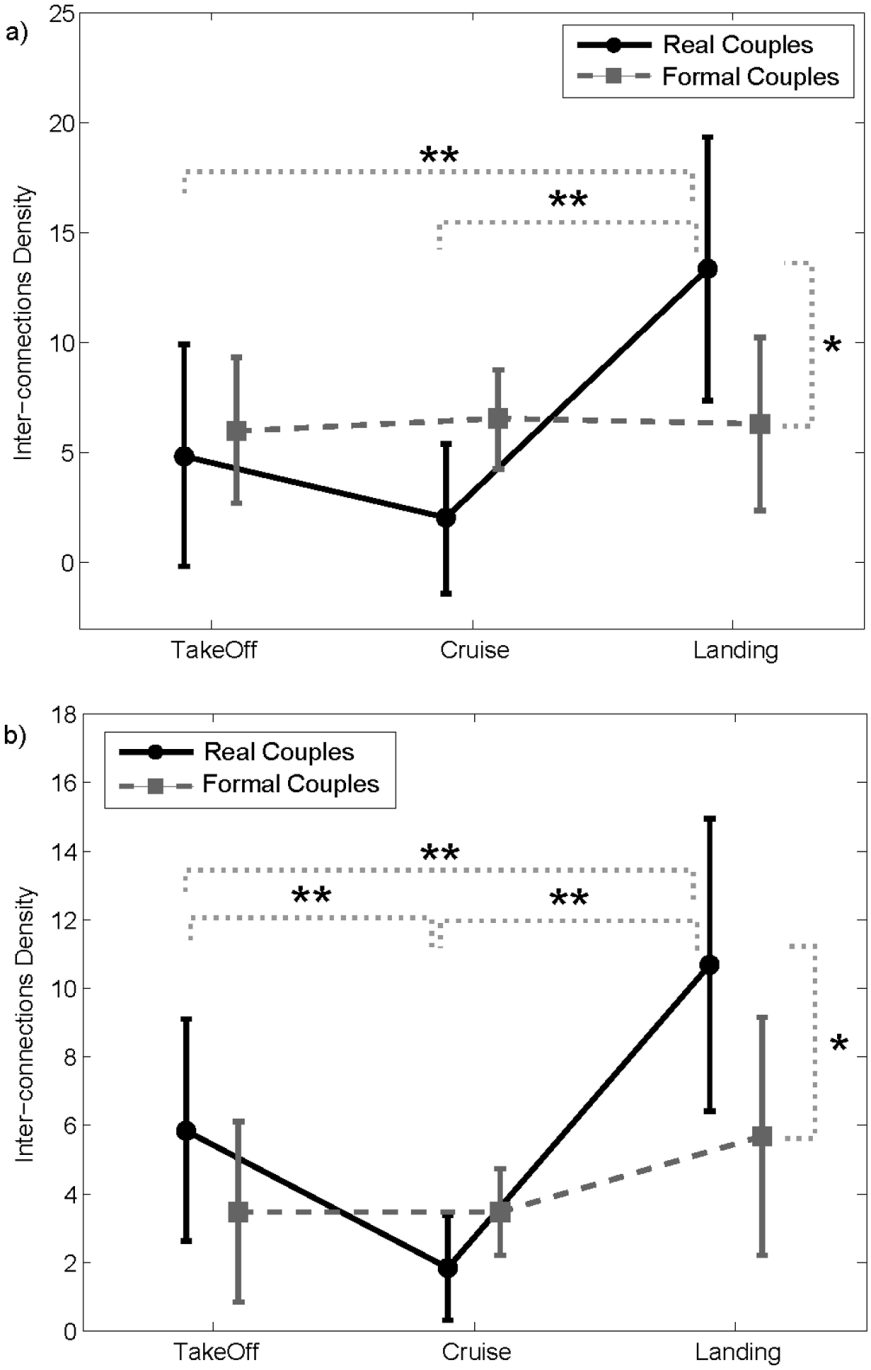
Graph Indices. Results of ANOVA performed on the *Interconnections Density* index computed on intersubject networks estimated in the theta (panel a) and alpha (panel b) bands, using FLIGHT-PHASE (takeoff, cruise and landing) and COUPLE-TYPE (real and formal) as the main *within* factors. The bars represent their relative 95% confidence intervals. The symbol (*) indicates the statistical difference between real and formal couples (Newman-Keuls’ pairwise comparisons). The symbols (**) indicates statistical differences between the flight phases for real couples (Newman-Keuls’ pairwise comparisons).

To better understand if the interbrain patterns obtained by the hyperscanning analysis were merely due to the common task performed by the two pilots, and if such analysis was able to capture the real interaction established between each specific pair, we also computed the interbrain connectivity analysis on “formal couples”, in which the pilots were randomly paired just according to their role, and not to the crew they were part of. In other words, we generated “formal crew” composed by a CPT and a FO who did not take part in the same flight simulation. We performed the same ANOVA described above, adding the COUPLE-TYPE (real and formal) between the main factor. The analysis revealed a statistically significant influence of the combined FLIGHT-PHASES x COUPLES-TYPE factor on the amount of interconnections between the two pilots in the theta (Fig 6a) (F = 4.49, p = 0.018) and alpha (Fig 6b) (F = 3.53, p = 0.046) bands. Newman-Keuls’ pairwise comparisons revealed significant differences between real and formal couples only in the landing phase for both the EEG bands. In such a flight phase, the Inter-connections Density was significantly higher in the real couples than in the formal ones. The significant difference between different flight phases found for the real couples was not confirmed for the formal couples.

**Fig 7:**
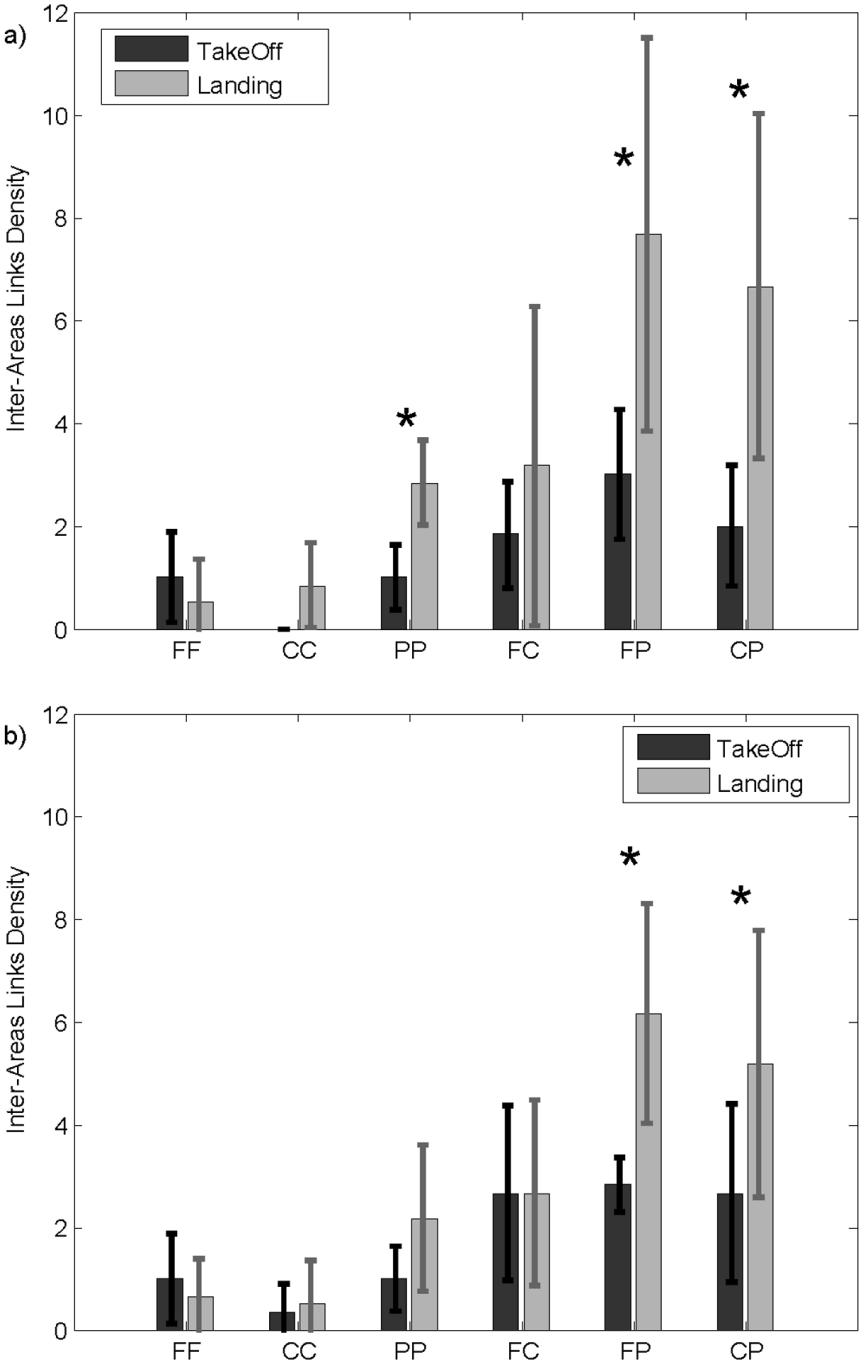
Inter-areas Links. Bar diagrams reporting the density of connections exchanged between brain macro-areas of the two crew members in the theta (panel a) and alpha (panel b) bands during takeoff (dark grey) and landing (light grey). The inter-areas links density reported here link frontal (FF), central (CC), and parietal (PP) areas of both pilots, as well as crossed connections linking frontal and central (FC), frontal and parietal (FP), and central and parietal (CP) brain areas. The symbol (*) indicates a statistical difference between the takeoff and landing phases (paired t-test, p<0.05).

To further investigate the cortical circuits underlying the interbrain patterns, we defined an index (*Inter-areas Links Density*) characterizing the connections between specific cerebral macro-areas in the two pilots in terms of the number of statistically significant paths linking those areas (frontal, central and parietal brain areas). As reported in Fig 7, for both the theta (panel a) and alpha (panel b) bands, the paired t-test revealed a higher density of fronto-parietal and centro-parietal interconnections exchanged between the two pilots during landing with respect to takeoff conditions. In the theta band, such an increase was also found for the parieto-parietal interconnections.

Given the different roles of the two pilots during the two cooperation phases, we investigated separately the interbrain connections density from CPT to FO and vice-versa.

**Fig 8:**
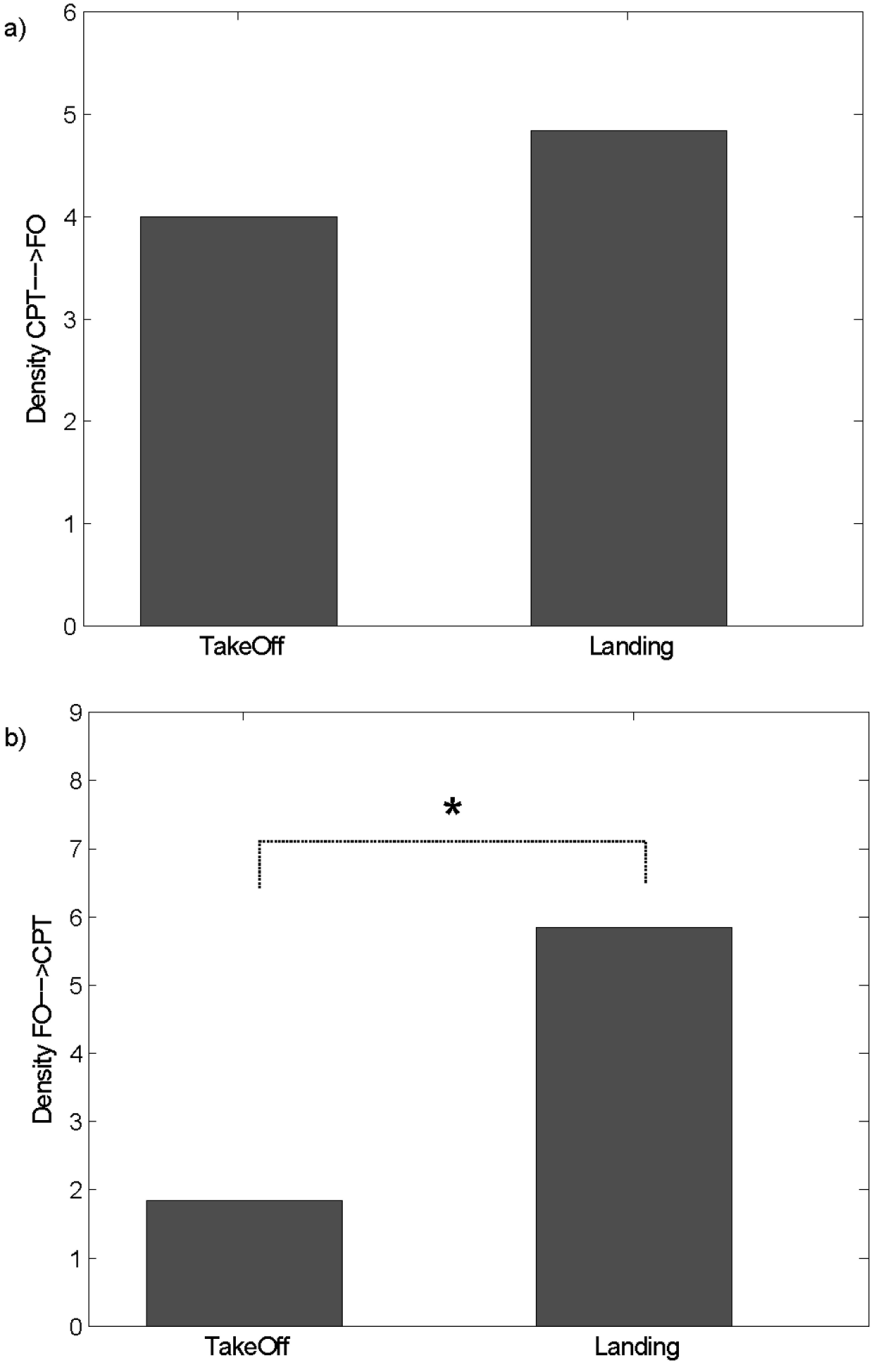
Inter-connections Directionality. Bar diagram reporting the density of connections directed from CPT to FO (panel a) and from FO to CPT (panel b) during the takeoff and landing phases in the alpha band. The symbol (*) indicates a statistical difference between the takeoff and landing phases (paired t-test, p<0.05).

We limited the analysis only to the two cooperative phases, where there was a high number of interconnections as described previously. In particular, we performed a paired t-test to compare the two cooperative phases, takeoff and landing, in terms of the density of the connections directed from the CPT to the FO (D_CPT→FO_) and vice-versa (D_FO→CPT_). No significant difference resulted for D_CPT→FO_ (Fig 8a) between takeoff and landing (paired t-test, p = 0.5646). Significantly higher values resulted, instead, for D_FO→CPT_ landing with respect to takeoff (Fig 8b) (paired t-test, p = 0.0472). No significant differences between the two phases resulted in the theta band for both of the indices.

## Discussion

The aim of the present study was to provide evidences that it is possible to detect neuroelectrical signs of brain activity related to the cooperative processes of a professional category (civil pilots) in an ecological setting. This was achieved by means of neuroelectrical hyperscanning and related intersubject connectivity analysis [18–20] during flight simulations involving twelve professional pilots (six couples) in a full-motion MD-80 simulator (Fig 1a) of the national Italian airline Alitalia. The flight mission was planned as to include in the simulation flight a framework of usual and unusual situations, which the pilots were trained to deal with. In fact, the introduction of an electrical failure to the CPT’s instrumentation led the crew to reorganize the mission (choose another airport destination) and their PF/PNF roles. A multi-modal approach, including advanced techniques for the analysis of different bio-signals, was adopted to investigate the psychological, peripheral and neuroelectrical aspects underlying the actions performed during a flight.

### Psychological and Peripheral Data

The analysis of psychological variables underlined an agreement between the construction of the experimental design and the pilots’ perception of the different flight phases. In fact, the electrical failures were inserted into the flight mission to increase the workload demanding along the flight (starting since the Taxi phase) and, consequently, to keep the pilots continuously engaged until the end of the landing phase, since the CPT had lost the control panel after the cruise phase and the FO had to take the control of the airplane. The analysis of the psychological data showed that the perceived mental workload were higher for the FOs than for the CPTs (Fig 3a). Moreover, both the pilot categories (Fig 3b) perceived the landing as the most difficult part of the mission. Therefore, the results highlighted that the electrical failure to the CPT’s instrumentation was successful in putting the crew in front of a more demanding task for the landing condition. In fact, the higher workload perceived by the FOs might be associated with the execution of unusual actions during the landing, while the higher frustration felt by the CPTs might be put in relation with the impossibility to use their instrumentation after the failure. The effect of the failure on the perceived workload was also confirmed by the analyses of the peripheral parameters. Moreover, a higher involvement of attentive processes (reduction of EBR) and a stronger emotive engagement (increase of HRindex) for the FOs with respect to CPTs during the landing confirmed what was found in the analysis of the psychological data [54].

### Brain Spectral Activations

In the takeoff, the FOs showed a significant increment of the theta activity in the frontal, parietal and occipital areas (Fig 4a), whilst no significant variations were found for the CPTs. In fact, during the takeoff phase, the CPT had the control of the airplane (PF) and the FO had to check the cockpit instrumentation (visual inputs) and report all the information (PNF) to the CPT. Such results were in accordance with the literature reporting that visual sensory inputs resulted in greater theta activity in the parietal and occipital brain areas [55,56] and when the task demand increased, the frontal theta increased as well [23]. In the landing phase, the occurrence of the electrical failure led instead to an increase of theta activity in the frontal areas of both pilots. Especially for the FOs, as they had to take the control of the airplane and to accomplish the procedures commensurate with the unusual flight situation. In addition, FOs showed high alpha synchronization over parietal areas in agreement with what was previously found for attention and working memory tasks [57,58].

### Single-subject Connectivity Patterns

The cognitive framework highlighted by the spectral brain mapping was confirmed by the single-subject connectivity analysis and the implementation of the related graph theory indices, which contributed to the neurophysiological characterization of the flight phases. Graph analysis revealed an efficient organization of the cerebral networks during takeoff and landing with respect to the cruise, as highlighted by higher values for both global and local efficiencies. Furthermore, these two flight phases, which were the most demanding ones, elicited denser networks than during the cruise, and were characterized by a higher involvement of the frontal cerebral areas. These results confirmed the higher cognitive engagement required during such demanding phases and were in line with the literature related to the field [59].

### Intersubject Connectivity Patterns

The results of single-subject connectivity, although able to track the pilots’ involvement in demanding flight phases, did not allow to capture specific brain signatures related to cooperation between the pilots. In fact, single-subject connectivity indices were able to characterize differences due neither to the level of interaction nor to the role within the crew.

To investigate the neurophysiological bases of cooperation between crew members, we used an extension of connectivity approach to the multi-subject case [19]. Connectivity patterns extracted for each pair showed a high number of significant information flows linking the two pilots during the most cooperative phases (takeoff and landing) of the simulated flight. Such interconnections broke down to nil during the cruise, in which the two pilots acted independently (Fig 5). The modulation of the amount of connections exchanged between the two pilots across the three different flight phases was confirmed by the ANOVA analysis on Inter-connections Density index (solid line in Fig 6). The index was able, in fact, to capture the different levels of cooperation between pilots by modulating its values across the three different phases. Such results were in line with previous hyperscanning studies on subjects’ pairs interacting in social contexts controlled by game theory [2]. Statistically significant connections were, in fact, observed between subjects during the cooperation condition, yet they were almost absent during the defect condition in the Prisoner Dilemma game [18]. Moreover, the density of such connections was demonstrated to be one of the potential predictors of cooperative behavior [20].

Results in Fig 6 also demonstrated the capability of the Inter-connections Density index to distinguish cerebral circuits associated with cooperative situations in which the two pilots were not recorded simultaneously (formal couples) from brain synchronizations related to an effective and simultaneous cooperative behavior (real couples). The modulation of such an index across the three different flight phases was, in fact, found only in the case of the real couples, where it was maximum during landing phase, high in takeoff phase and low during cruise. In the case of formal couples, the interbrain connections density was very low and not significantly different in cooperative and not cooperative situations. These results were in line with the literature reporting synchronizations between brain activities of subjects exposed to similar stimuli and not really interacting in a social context as the case of formal couples [60]. Formal couples represents a powerful baseline for avoiding the presence of spurious interbrain links due to the experimental design and not to a social behavior. The IBD was not null in the case of formal couples, as expected, but it represented the level of correlation between the brain activities of the two subjects only due to the environment and to the imposed protocol to be followed by the two pilots for succeeding in each flight phase. The IBD was significantly higher in real couples with respect to the formal ones and this revealed the importance of simultaneously recording the brain activity of two interacting persons. Only by means of a multivariate analysis of data simultaneously recorded, it was possible to achieve a description of the real interaction, depurated by all what is related to the exposure to common stimuli.

Moreover, the role of formal couples was crucial in the assessment of what we found in terms of intersubjects connectivity patterns since they represented an optimal baseline for the PDC estimator. It was noticed [61] how particular attention should be paid in adopting PDC for hyperscanning purposes in order to avoid false positives due to confound factors. In this paper, we confirmed the necessity to pay attention to this aspect as we found significant interbrain connections also in formal couples. However, we showed the added value provided by the simultaneous recording (real couples) with respect to offline pairing of subjects recorded in different moments (formal couples). In particular, when the two pilots were recorded simultaneously in a real cooperative situation (real couples), PDC estimator could capture the social interaction between them by providing a higher number of interbrain connections with respect to those provided in the control condition where pilots did not directly interact (formal couples).

Due to the electrical failure, the FO was forced to take the control of the aircraft, leading the two pilots in a situation out of the ordinary protocol adopted during the flight. In such situation, the pilots really had to cooperate and to find out the proper strategy to accomplish the mission. The landing phase represented the highest level of cooperation between pilots. During the takeoff, they had to collaborate but only by following a predefined protocol. This might explain the highest differences between real and formal couples in the landing phase, corresponding, as confirmed by psychological and peripheral data, to a compelling situation requiring a high degree of cooperation between the pilots.

It is well known how intersubject connectivity patterns might be affected by several factors: the connectivity estimator adopted [61], the statistical assessment of estimated values and an experimental design including appropriate baseline condition. To take into account the spurious links due to all these factors, we adopted a rigorous procedure including: i) the use of one of the most accurate and reliable connectivity estimator, the PDC [48], combined together with a valid procedure for the statistical assessment of estimated links [62], ii) the construction of an experimental design including a baseline condition, the taxi phase, to be used for removing differences in the amplitude of the power spectrum between the two pilots, iii) the use formal couples as control condition for the results achieved in interbrain connectivity, providing a baseline for PDC estimator and ensuring its ability in catching aspects of social behavior.

Once we investigated the main properties of the interbrain networks across the three different phases, we focused our attention on the two cooperative conditions; takeoff and landing. In particular, we first highlighted the brain macro-areas mainly involved in the interaction between pilots, and then investigated the presence of an imbalance in the number of connections exchanged between pilots due to their role.

Intersubject connectivity patterns detected in these two flight phases mainly linked the frontal and parietal brain areas of the two crew members, as confirmed by the *Inter-areas Links Density index* (Fig 7). These fronto-parietal connections were denser in the landing phase with respect to takeoff condition. Such results were also consistent with studies indicating how the pre-frontal and frontal areas, in particular the anterior cingulate cortex (ACC), represent the other’s intentions in the brain [63,64] having a prevalent role in decision-making processes, free task selection [65], formation of intentions [66], multitasking [67] and conflict monitoring [68]. Moreover, several hyperscanning studies highlighted the existence of robust synchronizations of the brain activities in the prefrontal, frontal and central parietal cortical regions during coordinated actions [9,69] or cooperative behaviors (Astolfi et al., 2011b).

The presence of a hierarchy in the pilots’ pairs allowed us to investigate the ability of the indicators extracted from interbrain networks in detecting the presence of a main direction in the connections exchanged between the two pilots. While the interactions directed from the CPT to the FO remained constant across the two flight phases, the interactions from FO to CPT increased significantly during the landing phase with respect to takeoff, as a consequence of the FO’s higher control of the aircraft given by the electrical failure induced to the CPT’s instrumentation (Fig 8).

Such study represents a proof a concept of the use of hyperscanning outside the lab, in a real operational context. However, due to the exceptional ecological environment used for the paradigm, the experimental group is limited to 6 couples of pilots since their recruitment required a lot of effort. We, thus, took into account the exiguity of experimental group in the choices made for data analysis. The main result of the manuscript, i.e. the modulation of multiple-brain connectivity patterns according to the different flight phases was provided for each pair (see Fig 5). We did not perform a group statistical analysis on connectivity patterns but we computed a single subject statistical analysis (higher statistical power) by validating the significance of each estimated connection against the chance level. The corresponding graph theory results were, instead, validated against those obtained on formal pairs, obtained by mixing the pilots based on their role (Fig 6). This led to increase the power of the statistical analysis.

## Conclusion

The methodology and the technology presented in this work were demonstrated to be employable in assessing cooperative states within the aircrew or more generic team members in ecological simulators and real working environments. Such results are not necessarily limited to the aeronautic field and could be extended and thus generalized to the whole social neuroscience context.

The novelty of the proposed study consisted of moving hyperscanning outside the lab and demonstrating its ability to track cooperative behaviors also in a real world scenario, such as the cockpit of an aircraft. Such study, even if conducted on a small sample of pilots, provided promising results and opening the way to the use of hyperscanning in real life social conditions.

Firstly, the hyperscanning approach and the related interbrain connectivity was demonstrated to be more sensitive to variations in the degree of interaction between subjects, or to changes in the interbrain patterns due to an effect of the hierarchy with respect to established methodologies for EEG signals processing applied at a single subject level. Spectral analysis and single-subjects connectivity estimations applied to EEG data recorded in challenging conditions (few electrodes, ecologic environment), provided rough information on the cerebral mechanisms underlying cooperation. Such approaches were not able to detect differences between different levels of interaction, nor to highlight the macro-areas mainly involved in cooperative behaviors. Only by considering the EEG signals of the two pilots as a unique dataset for interbrain connectivity estimations allowed us to increase the knowledge about those mechanisms.

Secondly, the statistical differences found between real and formal couples strongly demonstrated the necessity to simultaneously acquire EEG signals from two interacting subjects for investigating their cooperation. Formal couples modeled a cooperative condition in which the EEG data were not recorded simultaneously from the two pilots. Only the proposed combination of hyperscanning and interbrain connectivity allowed to capture brain networks corresponding to different levels of interaction between pilots.

The advancements proposed in this study allowed us to move a step forward in the exploitation of these approaches for the reconstruction of the brain circuits on the basis of social cognition.
